# Self-rated health, symptoms of depression and general symptoms at 3 and 12 months after a first-ever stroke: a municipality-based study in Sweden

**DOI:** 10.1186/1471-2296-8-61

**Published:** 2007-10-17

**Authors:** Ylva Skånér, Gunnar H Nilsson, Kristina Sundquist, Ejda Hassler, Ingvar Krakau

**Affiliations:** 1 CeFAM (Centre for Family and Community Medicine), Karolinska Institute, Alfred Nobels väg 12, SE-141 83 Huddinge, Sweden

## Abstract

**Background:**

Self-rated health is an important indicator of quality of life as well as a good predictor of future health. The purpose of the study was to follow up the self-rated health and the prevalence of symptoms of depression and general symptoms in a population of first-ever stroke patients 3 and 12 months after stroke.

**Methods:**

All patients surviving their first-ever stroke and residing in Nacka municipality in Stockholm County Council were included using a multiple overlapping search strategy during an 18-month period (*n *= 187). Our study group comprised the 145 patients who survived the first 3 months after stroke. Three and 12 months after their stroke, the patients were assessed regarding self-rated health and general symptoms using parts of the Göteborg Quality of Life Instrument (GQLI), and regarding symptoms of depression using the Montgomery Asberg Depression Scale (MADRS-S).

**Results:**

Self-rated health was rated as very good or rather good by 62% at 3 months after stroke and by 78% at 12 months after stroke. More than half of the patients suffered from symptoms of depression, with no significant improvement at 12 months. The most common general symptoms at 3 months after stroke were fatigue, sadness, pain in the legs, dizziness and irritability. Fatigue and sadness were still common at 12 months. Twelve months after stroke the prevalences of crying easily, irritability, impaired concentration, nausea and loss of weight were significantly lower.

**Conclusion:**

The majority of patients rated their health as rather good or very good at 3 and 12 months after stroke. However, the majority suffered from fatigue and from symptoms of depression after both 3 and 12 months. In continued care of stroke survivors, it is important to consider the fact that many patients who rate their health as good may nevertheless have symptoms of depression, and some of them may benefit from anti-depressive treatment.

## Background

Neurological handicaps and loss of functional independence are common after stroke, and are often associated with depression and a poor quality of life [1,2]. At some time after the onset of stroke one third of all those afflicted experience significant depressive symptoms [3]. Stroke patients can also be expected to suffer from a variety of general symptoms related to their perceived health. Patients' self-rated health is known to be an important indicator of their quality of life and it has also been shown to be a good predictor of their future health [4-9]. Several studies have shown a strong association between self-rated health and mortality [10-12]. Stroke has also been shown to be the chronic condition most strongly associated with poor self-rated health in individual patients [13]. When planning for rehabilitation and continued care after stroke, it is therefore important to recognize and deal with many factors in addition to the patient's functional status and neurological sequelae.

This study is part of a comprehensive cohort study of consecutively included first-ever stroke patients in Nacka, Sweden, that was carried out in order to document and enhance the medical care delivered to stroke patients [14]. In most population-based post-stroke studies, patients with communication difficulties like dementia and cognitive impairments are excluded from assessments of mood symptoms [3,15]. In our study we used validated instruments that have been used extensively and are easy to administer and fairly easy for patients to understand, and consequently no groups of patients were excluded. The survey therefore provides a comprehensive picture of the situation for first-ever stroke patients in a community. In this study we focus on patients' self-rated health, symptoms of depression and general symptoms.

The aim of this study was to investigate self-rated health and the prevalence of symptoms of depression and general symptoms in a population of first-ever stroke patients 3 and 12 months after stroke.

## Methods

### Setting

Nacka is a municipality of approximately 70 000 inhabitants (1999 figure) situated about ten kilometres east of Stockholm. We measured the socioeconomic characteristics of Nacka by means of the Care Need Index, which is an area-based socioeconomic index focusing on the distribution of primary health care resources. In the municipality of Nacka, the Care Need Index was 0.63, i.e. very close to the mean value for Sweden as a whole [16], which increases the probability of generalizing our findings. However, the population in Nacka was slightly younger than the general population in Sweden; 12% were 65 years or older in Nacka, compared to 17% in Sweden (1999), and the average age was 37.7 years compared to 40.3 years in Sweden as a whole (2000) (Statistics Sweden).

### Inclusion criteria and search strategies

Stroke was defined according to WHO criteria as a sudden focal disturbance of brain function with symptoms that persist for at least 24 hours, or which leads to death, and in which the cause is not obviously non-vascular. According to the International Classification of Diseases and Related Health Problems (ICD-10), this definition corresponds to the codes cerebral haemorrhage (I 61), cerebral infarction (I 63), and stroke (I 64). Patients with Transient Ischaemic Attack (TIA, I 65) were not included. Patients with subarachnoid haemorrhage (I 60) are included in some stroke studies, but because of differences regarding aetiology, risk factor pattern and management, they were not included in this study [2].

The study aimed at including all patients with a first-ever stroke who were registered as residing in Nacka at the onset of their illness. The inclusion period was 18 months (October 1999 – March 2001), and we prospectively included all patients, irrespective of age, co-morbidity or where the stroke had occurred. Thus several overlapping sources of case ascertainment were used, in accordance with recommendations for complete, community-based case ascertainment [17]. Since we planned to interview the patients 3 months after the stroke, it was our ambition to identify them as soon as possible, i.e. we were in "hot pursuit" of these patients [17]. A regular monthly search for new cases of first-ever stroke was carried out throughout the study period, and consisted of (1) searches in records at the radiology departments at both the regional hospital and its subsidiary at the municipal hospital for patients who had undergone any radiological investigation of the head (considered obligatory for stroke patients), (2) searches in patient records at the regional hospitals, including the emergency departments, (3) searches in discharge summaries from the geriatric department at the municipal hospital, (4) reports about new cases from contact persons in the project: two stroke nurses, a rehabilitation officer and an occupational therapist, (5) reports from managers of the health care centres in Nacka, who had knowledge about new cases of stroke at the nursing homes, and (6) a complementary search of the County Council's joint register of hospital care. Searches using strategy (1) were done by reading all relevant referral notes, and if any indications of stroke were found, by reading the medical records for further information; searches based on strategy (2) were carried out by reading the medical records of all newly admitted patients; searches based on strategy (3) were done by using ICD-10 diagnoses; searches using strategies (4) and (5) were conducted via personal contacts; and searches based on strategy (6) by use of ICD-10 diagnoses. Information from medical records as well as from patients and relatives was used to determine if the event found constituted a first-ever stroke.

Only three patients did not undergo computed tomographic (CT) or magnetic resonance (MR) imaging: two of them died within a few hours after hospital arrival, and one died within three days at the nursing home where he was being treated for colon cancer. The validity of the stroke diagnosis was always assessed by a senior neurology specialist.

The medical records of patients who fulfilled the inclusion criteria (*n *= 187) were obtained from medical, neurological, geriatric and rehabilitation departments, and were searched for information about co-morbidity and about impairments in consciousness, vision, language, and motor and sensory function. The information was extracted by one of the co-authors (EH), who is a general practitioner.

### The study group

Three months after their stroke, 42 patients were deceased and the 145 survivors constituted our study group. Twelve months after stroke there were 135 survivors. Since we wanted to describe self-rated health, symptoms of depression and general symptoms in the whole group of first-ever stroke patients, no exclusion criteria were used. Patients with cognitive impairments and/or aphasia and patients with severe physical morbidity were thus not excluded, as is the case in most studies of this kind [3]. Individual patients could, of course, choose not to participate or could be unable to participate.

### Instruments and outcomes

In order to study development of the outcomes during the first year, follow-up examinations were performed 3, 6 and 12 months after the stroke event. Two occupational therapists carried out three interviews with the patients in their homes, and assessed their functional level. The patients were first contacted by letter, and then one of the occupational therapists contacted the patient, or a relative, by telephone to arrange an appointment, which in most cases took place in the patient's home. The first interview was conducted as close as possible to 3 months after stroke (17 patients in the study group were found too late to be included in the 3-month investigation). Activities of Daily Living (ADL) was used as a global assessment of the patients' functional level, and ratings were done according to the criteria of Katz et al. (see Table 1) [18]. The patients were classified into seven categories, A-G, with A indicating that the patient had no need of help, and G that institutional care was necessary. Patients in categories A-C are generally considered to be functionally independent, requiring little or no help. The ADL level was assessed by a combination of information extracted from the medical records and information from the interviews.

**Table 1:** Katz ADL Index. The Katz ADL Index is used to assess functional status. Patients are rated on independence (+/-) in each of six basic activities (bathing, dressing, toileting, transferring, continence and feeding). A score of 6 (A) indicates full function and a score of 2-0 (E-G) indicates severe functional impairment [38].

	A	B	C	D	E	F	G
Bathing	+	-	-	-	-	-	-
Dressing	+	+	-	-	-	-	-
Toileting	+	+	+	-	-	-	-
Transferring	+	+	+	+	-	-	-
Continence	+	+	+	+	+	-	-
Feeding	+	+	+	+	+	+	-

Questionnaires were distributed for the assessment of the patients' 1) self-rated health, 2) symptoms of depression, and 3) general symptoms, and were filled in by the patients. On the three different occasions, three, four and two patients, respectively, received help from relatives in completing the questionnaires. In this study we chose to compare the patients' situation 3 and 12 months after stroke in order to reflect their short-term and long-term situations.

Self-rating of health was done using a part of the "Göteborg Quality of Life Instrument" (GQLI), which is an instrument for assessing health-related symptoms, and social, physical and mental well-being [19]. The GQLI is a validated instrument that has been used in a large number of studies [5,20,21]. The patients were asked to rate their overall health (self-rated health) on a five-step scale: very good, rather good, neither good nor poor, rather poor, or poor.

The prevalence of symptoms of depression was assessed by a self-rating version of the Montgomery Asberg Depression Rating Scale (Montgomery Asberg Depression Rating Scale Self-assessment, MADRS-S), which is an instrument for the assessment and follow-up of depression [22,23]. The MADRS-S instrument is published on the Internet and contains nine items (mood, feelings of unease, sleep, appetite, ability to concentrate, initiative, emotional involvement, pessimism, zest for life). Each item can be given 0–6 points on a Likert scale. The results are interpreted as follows: 0–11 points = no symptoms of depression, 12–20 points = symptoms of mild depression, > 20 points = symptoms of major depression, > 40 points = symptoms when hospitalization may be recommended [22]. Only patients who had responded to six or more items were included. For those who had responded to six, seven or eight items, the mean number of points for the questions they had responded to was used to impute values for the missing questions.

The prevalence of general symptoms was assessed by means of another part of the GQLI [5,19,24]. The subjects were asked to indicate whether or not they had experienced any of 30 specified symptoms within the previous 3 months. The symptoms covered six different symptom categories: mental, gastro-intestinal/urinary, musculoskeletal, metabolic, cardio-pulmonary and head/miscellaneous.

### Analyses

Between-group comparisons were made with Generalized Estimating Equations (GEE). In the GEE models, adjustment has been made for age and sex. Results are presented as Odds Ratios (OR) and *p*-values. Spearman's rank correlation was used for the assessment of associations between variables 12 months after stroke.

### Ethics

The research ethics committee of the Karolinska hospital approved the study (Reference no. 99–376).

## Results

### The study group

The study group consisted of 69 men (48%) and 76 women (52%). The mean (± SD) age of the group was 73.3 ± 11.8 years (median 77.3 years, range 36–92 years). A description of the study group is given in Table 2.

**Table 2:** Description of study population at time of stroke. Stroke-related discharge diagnoses, co-morbidity and other conditions of interest in the study population at time of stroke.

	Men *n *= 69	Women *n *= 76	All *n *= 145
DISCHARGE DIAGNOSES			
Ischaemic stroke	57 (83%)	56 (74%)	113 (78%)
Haemorrhagic stroke	3 (4%)	6 (8%)	9 (6%)
Non-specified stroke	9 (13%)	14 (18%)	23 (16%)
CO-MORBIDITY			
Hypertension	42 (61%)	34 (45%)	76 (52%)
Angina pectoris	13 (19%)	16 (21%)	29 (20%)
History of myocardial infarction	11 (16%)	2 (3%)	13 (9%)
Heart failure	9 (13%)	10 (13%)	19 (13%)
Atrial fibrillation/flutter	20 (29%)	20 (26%)	40 (28%)
Diabetes	22 (32%)	13 (17%)	35 (24%)
Dementia	2 (3%)	4 (5%)	7 (3%)
Cancer	6 (9%)	8 (11%)	14 (10%)
Alcohol abuse	12 (17%)	6 (8%)	18 (12%)
OTHER CONDITIONS			
Cognitive impairment	20 (29%)	23 (30%)	43 (30%)
Anti-depressant treatment	3 (4%)	5 (7%)	8 (6%)

### Response rates

The response rates are shown in Table 3. Of the 145 patients in the study group, 97 participated in both interviews, 29 in one interview and 19 did not participate in any interview. One reason for not participating in both interviews was when the patient had not been identified within 3 months after the stroke (17 patients). Other reasons were refusal to participate (18 patients), death (10 patients), emigration (1 patient), and no known reason (2 patients). There was no difference between the patients who participated in both the interviews and those who did not with respect to age, sex or presence of dementia and/or cognitive impairment.

**Table 3:** Response rates 3 and 12 months after stroke. Response rates for self-rated health, symptoms of depression and general symptoms 3 and 12 months after stroke.

ASSESSMENT	3 months (*n *= 145)	12 months (*n *= 135)
Self-rated health	105 (72%)	98 (73%)
Symptoms of depression^1)^	91 (63%)	96 (71%)
General symptoms^2)^	106 (73%)	97 (72%)

^1) ^At 3 months: 65 (71%) responded to nine questions, 11 (13%) to eight questions and 15 (16%) to six or seven questions. At 12 months: 77 (80%) responded to nine questions, 11 (11%) to eight questions, and 8 (8%) to six or seven questions.

^2) ^At 3 months, the number of non-responders to the questions about 30 symptoms varied from 38 (26%) to 42 (29%), with a median value of 27%. At 12 months, the number varied from 38 (28%) to 65 (48%), with a median value of 30%. There was a tendency at 3 months, and a strong tendency at 12 months, to respond to a lesser extent to the last questions (pain in the joints, backache, pain in the legs, sweating, difficulty urinating).

### ADL

The numbers of patients in the different ADL categories are shown in Table 4. After 12 months, no patient was in a higher ADL category than before the stroke, 104 patients (72%) were in the same ADL category, 31 patients (21%) were in a lower ADL category and 10 patients (7%) were dead.

**Table 4:** ADL before stroke and at 3 and 12 months after stroke. Number of patients in different categories of Activities of Daily Living (ADL) before stroke and at 3 and 12 months after stroke. Patients in categories A-C are functionally independent without help or with only little help (see Table 1).

ADL CATEGORY	Before stroke *n *= 145	3 months *n *= 145	12 months *n *= 135
A	113 (77.9%)	98 (67.6%)	92 (68.1%)
B	19 (13.1%)	14 (9.6%)	13 (9.6%)
C	8 (5.5%)	5 (3.4%)	9 (6.7%)
D	2 (1.4%)	2 (1.4%)	4 (3.0%)
E	1 (0.7%)	11 (7.6%)	7 (5.2%)
F	1 (0.7%)	11 (7.6%)	8 (5.9%)
G	1 (0.7%)	4 (2.8%)	1 (0.7%)

### Self-rated health

Table 5 summarizes the results regarding self-rated health, which was significantly better after 12 months than after 3 months (OR 2.02; *p *= 0.0019). Including the ADL level before stroke in the model did not change the odds ratio significantly. At 12 months after stroke there was a statistically significant association between the ADL Index and SRH (*p *= 0.020).

**Table 5:** Self-rating of health 3 and 12 months after stroke. Number of patients rating their health as very good, rather good, neither good nor bad, rather bad and bad 3 and 12 months after stroke.

SELF-RATED HEALTH	3 months (*n *= 105)	12 months (*n *= 108)
Very good	14 (13%)	21 (19%)
Rather good	51 (49%)	64 (59%)
Neither good nor bad	19 (18%)	16 (15%)
Rather bad	16 (15%)	5 (5%)
Very bad	5 (5%)	2 (2%)

Ninety patients took part in the assessments conducted 3 and 12 months after stroke. Fifty-two of the 60 patients who rated their health as very good or rather good 3 months after stroke still did so after 12 months. Nineteen of the 30 patients who rated their health as less good 3 months after stroke rated it as very good or rather good after 12 months.

### Symptoms of depression

Table 6 shows the prevalences of symptoms of depression at 3 and 12 months after stroke. There was no significant change regarding symptoms of depression between 3 and 12 months after stroke (OR 1.176; *p *= 0.45). Including the ADL level before stroke in the model did not change the odds ratio significantly. At 12 months after stroke there was a statistically significant association between symptoms of depression and ADL (*p *= 0.0045) and between symptoms of depression and SRH (*p *= 0.0071). The mean scores for each MADRS-S item 3 and 12 months after stroke are presented in Table 7. No single item seems to dominate the clinical picture.

**Table 6:** Prevalence of symptoms of depression 3 and 12 months after stroke. Prevalence of different levels of depression 3 and 12 months after stroke.

LEVEL OF DEPRESSION	3 months (*n *= 92)	12 months (*n *= 96)
No symptoms of depression (score < 12 points)	41 (45%)	39 (41%)
Symptoms of mild depression (score 12–20 points)	34 (37%)	49 (51%)
Symptoms of major depression (score > 20 points)	17 (18%)	8 (8%)

**Table 7:** Mean scores for each MADRS-S item. Mean scores for each MADRS-S item. The median value is 2 for Initiative at 12 months and 1 for all other items at 3 and 12 months.

MADRS-S item	3 months after stroke	12 months after stroke
Mood	1.64	1.43
Feeling of unease	1.83	1.71
Sleep	1.65	1.80
Appetite	1.64	1.45
Ability to concentrate	1.93	1.63
Initiative	1.99	1.93
Emotional involvement	1.68	1.44
Pessimism	1.58	1.50
Zest for life	1.50	1.40

During the first year after stroke, 27 patients were given antidepressant therapy for some period of time, and 7 of those patients had also received treatment before the stroke. Six of the 17 patients with symptoms of major depression 3 months after stroke and 4 of the 8 patients with symptoms of major depression 12 months after stroke were given antidepressant therapy at some time during the period (data not shown).

### General symptoms

**Figure 1:**
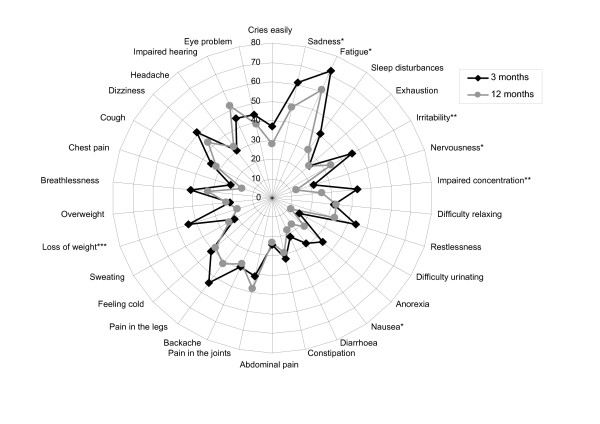
**General symptoms 3 and 12 months after stroke**. Prevalence (%) of general symptoms in the study group 3 and 12 months after stroke. For seven of the symptoms there is a significant difference between the situation at 3 months and at 12 months (* = *p *< 0.05, ** = *p *< 0.01, *** = *p *= 0.001). (The graph is a polar diagram, Excel, Microsoft™, see also [19])

Figure 1 shows the prevalence of general symptoms reported 3 and 12 months after stroke. At 3 months the most commonly reported general symptoms were fatigue (*n *= 72; 69%), sadness (*n *= 61; 58%), pain in the legs (*n *= 54; 52%), dizziness (*n *= 51; 48%) and irritability (49 patients; 46%). At 12 months the most commonly reported general symptoms were fatigue (61 patients; 58%), impaired hearing (52 patients; 49%), pain in the joints (48 patients; 48%), sadness (48 patients; 46%) and pain in the legs (42 patients; 45%). Twelve months after stroke the prevalence was significantly lower for the following symptoms: sadness (OR 0.61; *p *= 0.0299), fatigue (OR 0.60; *p *= 0.044), irritability (OR 0.54; *p *= 0.0179), nervousness (OR 0.0.48; *p *= 0.0343), impaired concentration (OR 0.43; *p *= 0.0010), nausea (OR 0.49; *p *= 0.0213) and loss of weight (OR 0.31; *p *= 0.002). Correlation for ADL level before stroke did not change the odds ratio significantly for any of the symptoms. At 12 months after stroke some symptoms were strongly associated with the MADRS-S scores, particularly fatigue (*p *= 0.0000), restlessness (*p *= 0.0001), sadness (*p *= 0.0001) and anorexia (*p *= 0.0020).

## Discussion

In this close to community-based study we investigated self-rated health, symptoms of depression and general symptoms in survivors of a first-ever stroke 3 and 12 months after the event. After 3 months, 62% of the patients rated their health as very good or rather good, and this was the case for 78% after 12 months. In another community-based follow-up study on first-ever stroke patients' self-rated health, 60% rated their health as excellent, very good or good 12 months after stroke [8]. However, these figures may not be directly comparable with ours, since the wording in our five-step scale for self-rating of health is symmetrical (middle value "neither good nor bad"), while their five-step scale is asymmetrical (middle value "good") which conforms to the wording in the first question in the quality of life assessment instrument SF-36[25]. When self-rated health among people aged 75 years or older was assessed by use of the GQLI in a Swedish epidemiological study, 87% of the responders rated their health as very good or rather good, which is significantly better than in our study [26].

Although the majority of patients were totally, or close to totally independent regarding ADL both before and after the stroke, 21% had a higher degree of dependence 12 months after stroke. Self-rated health was strongly associated with ADL, which corresponds to the findings in other studies, where, for example, the impact of ADL-dependency on quality of life for post-stroke patients was shown in a 2-year follow-up study where quality of life was assessed by a VAS-scale [27], and in a 3-year follow-up study where it was assessed by the SIP-instrument (Sickness Impact Profile) [28].

More than half of the patients in our study had symptoms of depression at some time, and there was no change in frequency between 3 and 12 months. In a recent meta-analysis study on frequency of depression after stroke that is based on 51 studies, six of which were population-based, the pooled average of the population-based studies was 33% (95% CI, 0% to 72%) in the early phase (1–5 months after stroke) and 34% (95% CI, 24% to 43%) 6 months or more after stroke [3]. Instruments for assessing depression differ, especially regarding patients classified as having symptoms of mild depression, and these patients may be included because of symptoms due to old age or physical diseases, and not depression. One observation from this meta-analysis study is that the highest frequencies of depression were found in the studies that used MADRS as the assessment instrument (pooled average 41%; 95% CI, 23% to 60%) [3]. Compared to other depression rating scales, MADRS-S has relatively fewer somatic items, and is thus less sensitive to concomitant physical illness [23]. This would be favourable in the present context, but it is probable that neurological and cognitive sequelae of stroke would nevertheless overlap with items in the MADRS-S scale. In a Swedish population-based study of depression in the oldest old (aged 85, 90 and 95+ years), the prevalence of any degree of depression, measured by the Geriatric Depression Scale-15 in combination with MADRS, was 26.9%. The 85-year-olds had a significantly lower prevalence than the two older groups (16.8% compared with 34.1% and 32.3%) [29].

Fatigue and sadness were among the most common symptoms at both 3 and 12 months after stroke. In another study, fatigue was reported by 68% of the patients 3 to 13 months after stroke, which corresponds well with our results [30]. In a recent two-year follow-up study of post-stroke fatigue in Sweden, 39% of the patients responded that they always or often felt tired, and this was associated with feelings of depression and deterioration in several aspects of everyday life [31]. The prevalences of general symptoms 12 months after stroke were surprisingly similar to the prevalences in a Swedish population (age range 16–92 years) [32]. The stroke patients had higher prevalences of anorexia, loss of weight, dizziness, impaired hearing, constipation, pain in the legs, sweating and breathlessness, a lower prevalence regarding headache, and about the same prevalences of fatigue and sadness. According to another Swedish study, some symptoms decrease with age (fatigue, abdominal pain, nausea, diarrhoea, cough and headache), while others increase (sleeping disturbances, pain in the joints, pain in the legs, breathlessness and impaired hearing) [24]. Most of the differences could thus be explained by the high age of the stroke patients.

Strengths of this study include the thorough, prospective search for patients, the absence of exclusion criteria, the use of a number of relevant outcome measures, and the use of everyday clinical records. It is thereby likely that these data provide a realistic picture of current practice in the area, which is sociodemographically representative of Sweden. However, it should be kept in mind that retrospective data from records only give information about what was actually recorded.

A limitation of the study is that the response rate for the interviews was rather low. One reason for this might be that patients with cognitive deficits, aphasia or severe handicaps were not excluded a priori in our study, and this may have influenced both the rate and quality of the responses. However, cognitive impairment or dementia were not more common among the patients who did not take part in the interviews and assessments at both 3 and 12 months after stroke than among those who did take part. The response rate was somewhat lower for the MADRS-S than for the GQLI. Our interpretation is that it was easier for patients to assess the presence or absence of symptoms (GQLI) than to use a rating scale to evaluate rather complex descriptions of symptoms and/or feelings (MADRS-S).

A potential limitation could be that despite our multiple search strategy, the search for cases might have been insufficient. However, there is a very strong tradition in Sweden and the other Nordic countries to refer patients suspected of having suffered a stroke to hospital for CT or MR imaging and, if possible, antiplatelet treatment. National, regional and local guidelines stress the importance of referring all patients with recent (less than a week) symptoms compatible with stroke or transient ischaemic attacks directly to hospital for further investigations and evaluation [33]. Recent Swedish studies using community-based stroke registers show that only about five percent of first-ever stroke patients have not been in contact with hospital, and these comprise mainly patients living in nursing homes [34-37]. General practitioners' intensified efforts over a period of time to refer all cases of stroke did not significantly increase the incidence rates [37]. Stroke patients whom we might have failed to include could thus be nursing home patients with concomitant severe diseases, or patients who did not see a doctor for their symptoms. However, because of the multiple overlaps in our search strategies, we are convinced that the study reflects the stroke situation in the community.

This study of first-ever stroke in a municipality provides a comprehensive picture of the well-being of these patients, who are often fragile in many respects and in need of different kinds of support. The clinical picture of these patients is complex and includes both neurological and less specific symptoms, and psychological symptoms may consequently be overlooked. To facilitate the continuity of patient care across different levels of care, it is important that doctors and other health care personnel not only communicate information about patients' neurological handicaps, treatments and co-morbidity, but also information about their moods, general symptoms, and worries and concerns about their own health. Many stroke patients suffer from fatigue and sadness at some time during the first year, and it is important to be observant in this regard and to investigate these findings further, since some patients may benefit from pharmacological treatment for depression. Further research is needed on the usefulness of these rating instruments in clinical settings.

## Conclusion

The majority of patients rated their health as rather good or very good at 3 and 12 months after stroke. However, more than half of the patients suffered from fatigue and from symptoms of depression after both 3 and 12 months. In continued care of stroke survivors, it is important to recognize and follow the development not only of patients' self-rated health, but also of their symptoms of depression and general symptoms. The clinical picture of these patients is complex, and many who rate their health as good may nevertheless have symptoms of depression, and some of them would benefit from anti-depressive treatment.

## List of abbreviations

ADL Activities of Daily Living

CT Computed tomographic imaging

GQLI Göteborg Quality of Life Instrument

MADRS Montgomery Asberg Depression Rating Scale

MADRS-S Montgomery Asberg Depression Rating Scale Self-assessment

MR Magnetic resonance imaging

OR Odds ratio

SD Standard Deviation

WHO World Health Organization

## Competing interests

The author(s) declare that they have no competing interests.

## Authors' contributions

IK and EH collected the original data. All authors have contributed to the conception and design of the study, the analyses and interpretation of the data, and the writing and approval of the final version of the manuscript.

## Pre-publication history

The pre-publication history for this paper can be accessed here:


